# Differences in anthropometric and vertical jump force-time characteristics between U16 and U18 female basketball players

**DOI:** 10.3389/fspor.2024.1425475

**Published:** 2024-06-25

**Authors:** Dimitrije Cabarkapa, Damjana V. Cabarkapa, Dora Nagy, Kenza Szabo, Laszlo Balogh, Sandor Safar, Laszlo Ratgeber

**Affiliations:** ^1^ Jayhawk Athletic Performance Laboratory—Wu Tsai Human Performance Alliance, Department of Health, Sport and Exercise Sciences, University of Kansas, Lawrence, KS, United States; ^2^ Faculty of Health Sciences, Doctoral School of Health Sciences, University of Pecs, Pecs, Hungary; ^3^ Faculty of Health Sciences, Institute of Physiotherapy and Sport Science, University of Pecs, Pecs, Hungary; ^4^ Center for Basketball Methodology of Education, Pecs, Hungary; ^5^ Institute of Sport Science, University of Debrecen, Debrecen, Hungary; ^6^ Training Theory and Methodology Research Center, University of Physical Education, Budapest, Hungary; ^7^ Department of Sport Games, Institute of Sports, University of Physical Education, Budapest, Hungary

**Keywords:** sport, performance, force, power, development, muscle, eccentric, concentric

## Abstract

Considering the importance of body composition and lower-body strength and power for basketball players' on-court performance, as well as a lack of sports science research focused on female athletes, the purpose of the present investigation was to record the anthropometric and countermovement vertical jump (CMJ) characteristics of top-tier U16 and U18 female basketball players and examine between-group differences in the aforementioned tests. Thirty-two athletes who were a part of the national basketball academy volunteered to participate in the present investigation. Following the body composition assessment conducted via a segmental multifrequency bioimpedance analyzer, athletes performed three CMJs while standing on a force plate system sampling at 1000 Hz. Independent *t*-test and Mann-Whitney *U*-test were used to examine between-group differences. The findings reveal significant differences in body composition and lower-body neuromuscular performance characteristics between female basketball players ages 16 and 18. Although no differences were observed in muscle and body fat percentages, the U18 group had significantly greater height, overall body mass (both muscle and fat mass), as well as greater segmental fat-free mass (trunk, both legs and arms), intracellular and extracellular water, and body mass index when compared to their U16 counterparts. On the other hand, the U18 group demonstrated longer eccentric, concentric, and braking phase duration, as well as overall contraction time when compared to the U16 players. In addition, the U18 athletes exhibited higher eccentric mean force and power, concentric impulse, peak power, and mean and peak force.

## 1 Introduction

Basketball is one of the most popular team sports worldwide. It is a fast-paced game that combines technical and tactical skills with repetitive sprinting, jumping, and change-of-direction movements, all separated by short periods of rest (1–5). Given its multifaceted nature, basketball requires players to possess adequate physical (e.g., height, body mass) and physiological characteristics (e.g., speed, strength, anaerobic and aerobic capacity) in order to achieve optimal performance (1, 5).

One of the most commonly performed movements in basketball, integrated into both defensive (e.g., blocking, rebounding) and offensive actions (e.g., passing, shooting) of the game, is the countermovement vertical jump (CMJ) (6). Previous research has shown that out of approximately 1,000 movements that basketball players perform during the game, 45 are jumping actions (6–8). Thus, as an integral skill related to determining a team's success, the CMJ is often included in the physical performance assessments of basketball players as a non-invasive and time-efficient strategy for examining athletes' lower-body neuromuscular performance characteristics (9–11). While various technologies (e.g., accelerometers, motion capture systems, jump mats) have been used for the CMJ analysis, force plate systems are considered a gold standard or criterion measure due to their in-depth evaluation of the jumping motion (12–15). Specifically, the force plate systems provide practitioners with not only the outcome metrics (e.g., jump height) but a plethora of kinetic and kinematic variables during both eccentric (ECC) and concentric (CON) phases of CMJ (12–15).

Previous research showed that CMJ performance significantly differs between ages (U14 vs. U18), sexes (male vs. female), positions (guards vs. forwards), playing time (starters vs. non-starters), and levels of competition (elite vs. collegiate) (4, 15–20). For example, when examining a cohort of male and female U14, U16, and U18 basketball players, significant improvements in CMJ performance were observed with an increase in age (4). Specifically, when compared to the U14 group, the U18 male and female basketball players had considerably greater vertical jump heights (males: 32.6 ± 6.4 vs. 38.2 ± 11.3 cm, females: 25.5 ± 3.9 vs. 33.2 ± 5.9 cm) (4). Also, the same investigation revealed that male athletes demonstrated significantly better CMJ performance (i.e., greater vertical jump height) when compared to their female counterparts (4). Moreover, considering the position-specific differences, in a recently published investigation, Cabarkapa et al. (16) showed that centers on a professional male basketball team exhibited significantly greater braking impulse, ECC mean force, and mean power in absolute terms, however, these differences dissipated once the aforementioned variables were normalized by athletes' body mass. Opposite results were observed during the CON phase of the CMJ, where centers demonstrated significantly lower relative CON mean and peak force when compared to the guards, while no differences were found in the absolute terms (16). While these findings offer valuable insights into the CMJ performance of basketball players, data concerning the female athlete population still remains limited. Thus, future research is warranted to better understand the neuromuscular performance characteristics of female basketball players, especially across different age groups.

Furthermore, another important factor directly related to successful performance in the game of basketball is body composition (21, 22). It has been shown that more successful players on the basketball team have higher skeletal muscle mass and lower body fat percentage, which can help them efficiently perform explosive actions during the game (e.g., vertical jumps and/or change of direction movements) and reduce their risk of overuse injuries (22–27). However, it should be noted that differences in body composition vary according to sex (male vs. female), level of competition (international vs. regional), as well as age (young vs. professional athletes) (22, 24). For example, due to differences in hormonal profiles, as well as biological requirements (e.g., pregnancy), females naturally tend to have a higher body fat percentage than males (24, 28). Also, as athletes mature, their body undergoes significant morphological changes at different time points over the years (24). Thus, it is imperative that practitioners frequently monitor changes in anthropometric characteristics, especially within the younger athlete population, as well as take into account the sex-related differences in body composition. This information can further help strength and conditioning professionals develop more individualized training programs that factor in the aforementioned discrepancies and morphological and physiological development (22).

Therefore, considering the importance of CMJ and body composition assessments for basketball players, as well as a lack of sports science research focused on female athletes, the purpose of the present investigation was to record the anthropometric and CMJ characteristics of U16 and U18 female basketball players and examine if there are any significant differences in the aforementioned tests between the groups.

## 2 Materials and methods

### 2.1 Participants

Thirty-two female basketball players who were a part of the national academy volunteered to participate in the present investigation, from which fifteen athletes were competing at the U16 and seventeen at the U18 level of competition. All athletes were cleared by their respective sports medicine staff to participate in team activities. No athlete reported any kind of musculoskeletal injuries that could potentially limit or impair testing procedures. The testing procedures performed in this investigation were conducted in accordance with the Declaration of Helsinki.

### 2.2 Procedures

The testing procedures performed in the present study entailed body composition assessment followed by CMJ lower-body neuromuscular performance analysis. The testing was conducted on the same day during the pre-season competitive period as a part of mandatory evaluations administered by the national basketball academy. The same group of sports scientists and strength and conditioning practitioners completed all testing procedures. Both groups of athletes were tested at the approximately same time of the day (i.e., 11:00–14:00 h).

The body composition was assessed via a segmental multifrequency bioimpedance analyzer (InBody 770, Seoul, South Korea). The validity and reliability of this system has been previously documented (29–32). Athletes were instructed to wear light clothing (e.g., compression shorts and shirts), remove shoes and socks, and step barefoot on the device (i.e., feet positioned in line with the electrodes). When prompted, athletes grabbed hand electrodes (i.e., one in each hand) by placing the thumbs on the thumb electrodes and wrapping the fingers around the bottom electrodes. Then, they were instructed to keep the arms relaxed slightly away from the body (i.e., no contact between the arm and torso—15 deg shoulder abduction) and stay calm for 60 s. Prior to the start of the testing procedures, the following guidelines were provided to the participants to optimize the accuracy of the results: (a) no intense exercise 6 h before the testing; (b) no eating 2 h before the testing; (c) no drinking any type of fluid 30 min before the testing; (d) no alcohol or caffeine consumption 24 h before the testing; (e) avoid using body lotion or ointment on hands and feet before testing. Both feet and hand electrodes were cleaned and dried between each participant. The detailed description of variables obtained from the bioimpedance analyzer is presented in Table 1.

**Table 1:** List and definition of body composition variables examined in the present study.

Body composition variables [unit]	Definition
Height [cm]	Distance from the bottom of the feet to the top of the head.
Body mass [kg]	Total body mass including both muscle and fat mass.
Muscle mass [kg]	Total amount of skeletal muscle in the body.
Body fat mass [kg]	Total amount of body fat (surface level and internal fat).
Muscle percentage [%]	Ratio of muscle mass divided by total body mass.
Body fat percentage [%]	Ratio of fat mass divided by total body mass.
Fat-free mass right leg [kg]	Amount of all the non-fat components in the right leg.
Far-free mass left leg [kg]	Amount of all the non-fat components in the in the left leg.
Fat-free mass right arm [kg]	Amount of all the non-fat components in the right arm.
Fat-free mass left arm [kg]	Amount of all the non-fat components in the left arm.
Fat-free mass trunk [kg]	Amount of all the non-fat components in the trunk.
Intracellular water [L]	Body water inside cells.
Extracellular water [L]	Body water outside cells.
Body mass index [kg/m^2^]	Body mass divided by the square of the body height.

The CMJ testing was completed on a dual uni-axial force plate system (ForceDecks Max, VALD Performance, Brisbane, Australia) sampling at 1,000 Hz. Each athlete completed three non-consecutive CMJ without an arm swing (i.e., hands on the hip during the entire movement). The mean value across three jump trials was used for performance analysis purposes. To minimize fatigue-induced performance changes, each jump was separated by a 10–15 s rest interval. The system was recalibrated between each participant and research assistants were present to constantly provide strong verbal encouragement. The athletes were instructed to step on the force plate, quickly drop into a squat position at a self-selected depth, and explosively without pausing push the ground to spring back up into a maximal-effort vertical jump (33). Based on previously published research reports that demonstrated high practical applicability and solid levels of validity and reliability, the CMJ performance analysis included force-time metrics within both the ECC and CON phases of the movement (12, 15, 34, 35). The ECC phase represented a portion of the CVJ containing negative velocity. The CON phase started at zero velocity and ended at the time point of take-off. The breaking phase was a sub-phase within the ECC portion of the jumping movement, starting at minimum force until the end of the ECC phase. The detailed definition of force-time metrics examined in this study can be found in the VALD user manual (https://support.vald.com/hc/en-au).

### 2.3 Statistical analysis

Descriptive statistics, mean (standard deviation) or median (interquartile range), were calculated for each dependent variable examined in the present investigation. Shapiro-Wilk's test was used to assess if the assumption of normality was violated. For the variables that violated the assumption of normality (i.e., muscle mass, intracellular water, extracellular water, and eccentric peak power) the Mann-Whitney *U*-test was used to examine statistically significant differences between U16 and U18 female basketball players. On the other hand, for the remaining anthropometric and CVJ variables that did not violate the assumption of normality, the independent *t*-test was used. Effect sizes were quantified using Cohen's d (0.2 small, 0.5 moderate, >0.8 large effect) for normally distributed data and Wilcoxon's r (<0.3 small, 0.3–0.5 moderate, ≥ 0.5 large effect) for non-normally distributed data. Statistical significance was set *a priori* to *p* < 0.05. All statistical analyses were completed with SPSS (Version 26.0; IBM Corp., Armonk, NY, USA).

## 3 Results

Descriptive and comparison statistics for each dependent variable examined in the present investigation can be found in Tables 2, 3. While no between-group differences in muscle and body fat percentage have been observed, the results reveal that U18 basketball players are taller and heavier than U16 players. Also, U18 players have greater muscle and body fat mass, fat-free mass for the trunk, right and left leg, right and left arm, intracellular and extracellular water, as well as greater body mass index than their U16 counterparts (Table 2).

**Table 2:** Body composition and measurements and comparison statistics between U16 and U18 female basketball players.

Variable	U16	U18	*p*-value	ES
Height [cm]^b^	172.3 ± 6.8	179.0 ± 7.9	0.016	0.909
Body mass [kg]^b^	59.9 ± 7.6	70.2 ± 9.2	0.002	1.221
Muscle percentage [%]	45.7 ± 2.1	45.3 ± 1.6	0.517	0.214
Body fat percentage [%]	17.3 ± 3.5	19.0 ± 2.6	0.134	0.551
Muscle mass [kg]^a,b^	27.7 (7.4)	31.7 (6.2)	0.006	0.480
Body fat mass [kg]^b^	10.5 ± 2.6	13.3 ± 2.7	0.005	1.056
Fat-free mass right leg [kg]^b^	8.08 ± 1.21	9.09 ± 1.38	0.036	0.778
Fat-free mass left leg [kg]^b^	8.05 ± 1.23	9.10 ± 1.40	0.034	0.797
Fat-free mass right arm [kg]	2.4 ± 0.4	2.8 ± 0.5	0.005	0.883
Fat-free mass left arm [kg]	2.4 ± 0.4	2.8 ± 0.4	0.009	1.000
Fat-free mass trunk [kg]	21.0 ± 2.5	23.8 ± 2.5	0.005	1.120
Intracellular water [L]^a,b^	22.8 (5.7)	25.8 (4.8)	0.006	0.474
Extracellular water [L]^a,b^	14.4 (2.9)	15.5 (2.9)	0.018	0.417
Body mass index [kg/m^2^]^b^	20.1 ± 1.39	21.7 ± 1.40	0.004	1.147

AU, arbitrary units; ES, effect size.

^a^ non-normally distributed variable.

^b^ statistically significant difference between the groups (*p* < 0.05).

**Table 3:** Vertical jump force-time metrics and comparison statistics between U16 and U18 female basketball players.

Variable	U16	U18	*p*-value	ES
Braking phase duration [s]^b^	0.254 ± 0.032	0.288 ± 0.034	0.006	1.030
ECC braking impulse [Ns]	44.9 ± 12.1	48.7 ± 6.5	0.285	0.391
ECC breaking impulse/BM [Ns/kg]	0.747 ± 0.165	0.700 ± 0.087	0.321	0.356
ECC duration [s]^b^	0.426 ± 0.042	0.470 ± 0.035	0.003	1.138
ECC peak velocity [m/s]	−1.3 ± 0.2	−1.3 ± 0.3	0.895	0.000
ECC mean force [N]^b^	589.9 ± 74.4	690.5 ± 89.9	0.002	1.219
ECC mean force/BM [N/kg]	9.8 ± 0.2	9.8 ± 0.1	0.963	0.000
ECC peak force [N]	1,409.5 ± 180.7	1,546.8 ± 235.4	0.077	0.654
ECC peak force/BM [N/kg]^b^	23.5 ± 2.2	22.4 ± 3.1	0.085	0.404
ECC mean power [W]^b^	387.3 ± 90.6	450.1 ± 68.7	0.034	0.781
ECC mean power/BM [W/kg]	6.5 ± 1.2	6.5 ± 0.9	0.993	0.000
ECC peak power [W]^a^	1,338.0 (557.0)	1,336.0 (266.5)	0.455	0.480
ECC peak power/BM [W/kg]	21.2 ± 5.2	19.5 ± 3.1	0.282	0.397
CON duration [s]^b^	0.255 ± 0.032	0.283 ± 0.029	0.013	0.917
CON impulse [Ns]^b^	137.2 ± 15.1	157.0 ± 21.8	0.006	1.056
CON impulse/BM [Ns/kg]	4.8 ± 0.4	5.0 ± 0.4	0.117	0.500
CON peak velocity [m/s]	2.4 ± 0.3	2.4 ± 0.2	0.604	0.000
CON mean force [N]^b^	1,129.1 ± 116.6	1,245.7 ± 163.8	0.029	0.820
CON mean force/BM [N/kg]^b^	18.9 ± 1.7	17.8 ± 1.0	0.007	0.789
CON peak force [N]^b^	1,409.1 ± 160.6	1,558.6 ± 222.1	0.039	0.771
CON peak force/BM [N/kg]^b^	23.6 ± 2.1	22.2 ± 1.7	0.049	0.732
CON mean power [W]	1,472.8 ± 132.1	1,589.6 ± 261.0	0.077	0.565
CON mean power/BM [W/kg]	24.4 ± 2.2	22.7 ± 2.9	0.074	0.660
CON peak power [W]^b^	2,627.8 ± 264.8	2,926.6 ± 443.8	0.030	0.818
CON peak power/BM [W/kg]	44.1 ± 3.5	41.8 ± 5.0	0.159	0.533
Contraction time [s]^b^	0.681 ± 0.063	0.753 ± 0.058	0.002	1.189
RSI-modified [ratio]	0.425 ± 0.072	0.385 ± 0.062	0.104	0.595
Jump height [cm]	27.0 ± 3.9	25.9 ± 4.8	0.463	0.251

ECC, eccentric; CON, concentric; BM, body mass; ES, effect size. RSI, reactive strength index.

^a^ non-normally distributed variable.

^b^ statistically significant difference between the groups (*p* < 0.05).

When comparing between-group differences in lower-body neuromuscular performance characteristics, U18 players revealed notably longer braking phase duration and overall contraction time, ECC duration, ECC mean force, ECC mean power, CON duration, CON impulse, and CON peak power. In addition, CON mean and peak force were greater for U18 players, while CON mean and peak force/BM were slightly lower within the game group of athletes when compared to their U16 counterparts (Table 3).

## 4 Discussion

The present investigation aimed to examine the differences in the anthropometric and neuromuscular performance characteristics between U16 and U18 female basketball players, revealing several important findings. First, it was found that the U18 group had significantly greater height, overall body mass, including both muscle and fat mass, as well as greater segmental fat-free mass (i.e., trunk, both legs and arms), intracellular and extracellular water, and body mass index when compared to their U16 counterparts. However, no significant differences were observed in muscle and body fat percentages. On the other hand, when examining lower-body neuromuscular performance characteristics, it was found that the U18 group of basketball players demonstrated longer braking phase duration, ECC and CON durations, as well as overall contraction time when compared to the U16 players. Also, the U18 group exhibited higher ECC mean force and power, CON impulse, peak power, and mean and peak force, while the U16 group displayed a slightly higher relative CON mean and peak force.

The findings pertaining to the anthropometric characteristics of U16 and U18 female basketball players seem to closely align with the ones reported in previous research investigations (36, 37). For example, Drinkwater et al. (36) showed that as male and female basketball players mature (i.e., 14–19 years), their height and body mass tend to increase, with a small plateau occurring at age 17. Similar observations were made in the present study where U18 female basketball players had significantly greater height and overall body mass when compared to the younger group (i.e., U16). However, despite a significant increase in fat mass, as well as total and segmental fat-free mass, athletes’ muscle and body fat percentages remain relatively constant over the span of two years. In other words, while athletes appeared to gain more muscle over time, they also gained fat, which ultimately resulted in attaining similar muscle and fat proportions. These differences in body composition between the U16 and U18 groups of basketball players can be explained by the natural physical and hormonal changes that occur in females during adolescence (38, 39), as well as by the increase in training loads that often take place at the end of puberty (22, 40). Moreover, our results indicated that as athletes get older, their total body water significantly increases, including both intracellular and extracellular water, which can be largely attributed to the previously discussed increase in overall lean mass (41). However, while these findings offer a deeper insight into the anthropometric characteristics of female basketball players, there is still a lack of scientific literature focused on examining female athletes, especially during the adolescent period, thus further research on this topic is warranted.

To the best of our knowledge, this is the first investigation that examined the changes in neuromuscular performance characteristics between U16 and U18 female basketball players, during both ECC and CON phases of the jumping motion. First, it can be observed that the U18 group had significantly greater braking, ECC, and CON phase durations when compared to the U16 group. The physical maturation, neuromuscular development, as well as specific training adaptations that occur within this two-year time frame may be the primary contributors to the aforementioned discrepancies (22, 36, 40). Specifically, as athletes gain more muscle mass and experience with training over the years, their neuromuscular performance significantly improves, ultimately resulting in better movement control and timing of muscle contractions (42, 43). On the other hand, it is interesting to note that the braking, CON, and ECC phase durations (i.e., 0.29 ± 0.03, 0.47 ± 0.03, and 0.28 ± 0.02 s, respectively) obtained for U18 female basketball players in this study are similar to the ones detected for the elite female volleyball athletes (i.e., 0.29 ± 0.06, 0.49 ± 0.65, and 0.28 ± 0.03 s, respectively) who were part of the two-time world champion National Team (18). Thus, it can be concluded that the U18 group possesses similar CMJ strategies and mechanics as some of the top-tier female athletes in the world (18). Also, these findings may indicate that from age 16 to 18, athletes examined in the present investigation have undergone effective training programs that helped them significantly improve their jumping movement mechanics and efficiency, which ultimately resulted in superior CMJ performance.

When examining the body mass-dependent variables (i.e., impulse, mean and peak force, and power) during the ECC phase of the jumping motion, differences between the U16 and U18 groups have been detected only in absolute terms. However, once the body mass was taken into consideration (i.e., relative terms), both groups seemed to display similar performance characteristics. These findings are comparable to the ones obtained by Cabarkapa D et al. (16), where centers on a professional male basketball team had significantly greater absolute ECC mean force and power than the guards and forwards, while no differences were observed in the same force-time metrics expressed in relative terms. Furthermore, during the CON phase of the CMJ, the U18 group of female basketball players had significantly greater absolute mean and peak force and peak power, when compared to their U16 counterparts. However, once adjusted by body mass, the opposite trend could have been observed in the aforementioned variables. Similar findings were obtained by Cabarkapa et al. (16) where centers had greater body mass and absolute concentric mean and peak force (2,040 ± 157 and 2,497 ± 150 N, respectively) when compared to forwards (1,938 ± 224 and 2,409 ± 362 N, respectively), but their relative values were lower (18 ± 0.5 vs. 21 ± 2 and 22 ± 0.7 vs. 26 ± 3 N/kg, respectively). While further research is warranted on this topic, these findings can be explained by the fact that the increase in athletes' body mass from age 16 to 18 (∼10 kg) may have been greater than the increase in their force and/or power production (∼100 N or ∼200 W), thus causing a decrease the in previously mentioned force-time metrics expressed in relative terms.

Lastly, the U16 and U18 groups examined in the present investigation exhibited similar performance on the outcome metrics (e.g., jump height). These findings align with previous research showing that the jump height of female basketball players did not differ significantly between the U16 and U18 groups during the squat jump, as well as CMJ with and without an arm swing (44). Similar findings were observed among female handball players where the U16 group did not have significantly different jump heights when compared to their U18 counterparts (45). Thus, relying solely on observing and analyzing the outcome metrics such as vertical jump height, may not provide practitioners with a comprehensive understanding of athletes' CMJ characteristics and may limit and impair the overall interpretation of the impact of the maturation process and development (35, 46). In addition, it should be noted that RSI-modified was not significantly different between U16 and U18 female basketball players. While further research on this topic is warranted, especially on young female athletes, U16 players may not have shown significant differences in RSI-modified when compared to their U18 counterparts because they are still in the early stages of development and have not yet reached the level of neuromuscular efficiency and physical maturity that older players already possess such as seen by McMahon et al. (47) who focused on examining mature professional male rugby players.

While the present study provides sports practitioners with a deeper insight into the anthropometric and neuromuscular performance characteristics of U16 and U18 female basketball players, it is not without limitations. First, both assessments were performed only once during the pre-competitive period. Also, position-specific differences were not analyzed in the present study. Thus, future research should try to examine if these findings remain consistent across the competitive season (e.g., mid-season and post-season) and different basketball playing positions (e.g., centers, forwards, guards). In addition, future research should examine how other factors such as sleep, nutrition, and travel impact the aforementioned findings.

## 5 Conclusion

In conclusion, the findings of the present study reveal that female basketball players experience significant changes in body composition and CMJ neuromuscular performance characteristics between ages 16 and 18. Thus, it is important for sports practitioners to consider these differences and adjust their training regimens according to athletes’ status of maturation. In addition, the results for body composition and CMJ metrics obtained in the present investigation can provide practitioners with reference values, which can be used during the assessment protocols to determine further areas for improvement.

## Data Availability

The raw data supporting the conclusions of this article will be made available by the authors, without undue reservation.
